# Anatomical Considerations of Intramedullary Humeral Nailing and Lengthening

**DOI:** 10.3390/jcm9030806

**Published:** 2020-03-16

**Authors:** Gilbert Manuel Schwarz, Lukas Zak, Lena Hirtler, Gerald Eliot Wozasek

**Affiliations:** 1 Department of Orthopedics and Trauma-Surgery, Medical University of Vienna, Währinger Gürtel 18-20, A-1090 Vienna, Austria; lukas.zak@meduniwien.ac.at (L.Z.); gerald.wozasek@meduniwien.ac.at (G.E.W.); 2 Division of Anatomy and Cell Biology, Medical University of Vienna, Währinger Straße 13, A-1090 Vienna, Austria; lena.hirtler@meduniwien.ac.at

**Keywords:** humerus, intramedullary lengthening, medullary cavity, cortical thickness

## Abstract

Intramedullary lengthening, in cases of extensive humeral shortening, offers the advantages of preventing external-fixator-associated problems. The humeral cavity, as the main parameter in nailing, however, has been neglected in recent literature. It was hypothesized that available implants might be too large and therefore increase the risk of intraoperative fractures. The aim of this cross-sectional study was to describe the humeral canal and how it might affect the choice of implant and the surgical approach. Thirty humeri (15 female, 15 male) from clinical patients and anatomical specimens were studied. Specifically, the medullary cavity width (MCW), cortical thickness (CoT), and the course of the medullary canal were examined. The smallest MCW diameters were found at the distal third of the humeral shaft with mean diameters of 10.15 ± 1.96 mm. CoTs of female humeri were significantly smaller than those of male humeri (*p* < 0.001). The mean angles of the pro- and recurvatum were 4.01 ± 1.68° and 10.03 ± 2.25°, and the mean valgus bending was 3.37 ± 1.58°. Before implanting a straight lengthening nail into a doubly curved humerus, X-rays and, in selected cases, CT-scans should be performed. The unique size and course of the humeral canal favors an antegrade approach in cases of intramedullary lengthening.

## 1. Introduction

Limb lengthening goes back to the work of Ilizarov, who initially introduced an external apparatus for bone fragment fixation and lengthening in the early 1950s [1]. External fixation of bones, however, carries the risk of pin infection, perioperative nerve and soft tissue irritation, and discomfort during treatment [2]. Therefore, intramedullary nailing and distraction was introduced. 

In 2015, an expert review depicted intramedullary nailing and lengthening for the humerus [3]. Patient satisfaction, disability, and functionality scores showed improvements with these interventions [4,5,6,7]. A similar consolidation index to external lengthening was also seen [8]. The main advantage of intramedullary implants is that they ensure Range-of-Motion (ROM) during the lengthening phase at pre-surgery levels [6].

There is scarce literature regarding complications in lengthening with straight intramedullary implants [6]. In fracture treatment, the most feared complications are iatrogenic supracondylar fractures during retrograde nail insertion [9]. Detailed knowledge of the humeral anatomy and exact fitting implants are essential to avoid this complication [4]. In 1944, Böhler et al. showed the unique S-shaped curvature of the humeral canal, and recent studies have reinforced these observations [10,11]. Significant differences regarding the minimal cavity diameters and cortical bone thickness (CoT) among ethnicities have been demonstrated. CoT in patients of South Asian descent, for example, is larger than that observed in patients of Arab descent, and as a result, the medullary cavity is narrower [12]. Consequently, not all humeri can be treated with the same nail size, and different implants should be used according to the given anatomical conditions. This applies even more to patients with post-traumatic or congenital humeral shortening [5].

It was hypothesized that available implants might be too large and therefore increase the risk of intraoperative fractures during retrograde nail insertions. The purpose of this study was to describe the anatomy of the humeral canal and its clinical relevance in fitting a straight nail down the doubly curved humeral bone.

## 2. Material and Methods

Thirty humeri (15 female, 15 male) from clinical patients and anatomical specimens were evaluated in this cross-sectional study. The study was approved by the Human Ethics Committee and the institutional review board of the Medical University of Vienna (Nr. 2241/2018). This manuscript is structured according to the STROBE checklist [13]. All specimens were of Caucasian origin. Although samples were randomly selected, special focus was placed on choosing the same numbers of female and male humeri. Different examination modalities were applied to investigate the special 3-dimensional shape of the humeral cavity, including its S-shaped pro- and recurvatum as well as valgus bending. Including patients with preexisting CT imaging allowed for the examination of humeri in a younger population, addressing a potential selection bias. All CT and radiographic measurements were performed using IMPAX EE (2018 Agfa-Gevaert Group, Mortsel, Belgium) and ImageJ software 1.50e [14]. Specimens were subdivided into two groups depending on the different origins of the humeri:

- Group 1—patients with CT imaging of the humerus (*n* = 10)
  o 3 female humeri, 7 male
- Group 2—anatomical specimens (*n* = 20)
  o 12 female humeri, 8 male

### 2.1. Group 1 CT Evaluation (10 Humeri)

CT images from 7 male and 3 female humeri were retrospectively retrieved from polybody scans performed at our clinical department for trauma surgery from September to November 2018. The CT device used was the SOMATOM Edge plus (SIEMENS Healthineers, Erlangen, Germany). Each humerus was reconstructed in the coronal, sagittal, and axial planes, and measurements were taken from the surgical neck of the humerus to the supracondylar fossa. The humeral cavity was divided into 7 segments, and axial images were used for sagittal and coronal measurements. For each level, detailed anterior, posterior, medial, and lateral CoT measurements were made.

### 2.2. Group 2 Anatomical Evaluation (20 Humeri)

**Figure 1:**
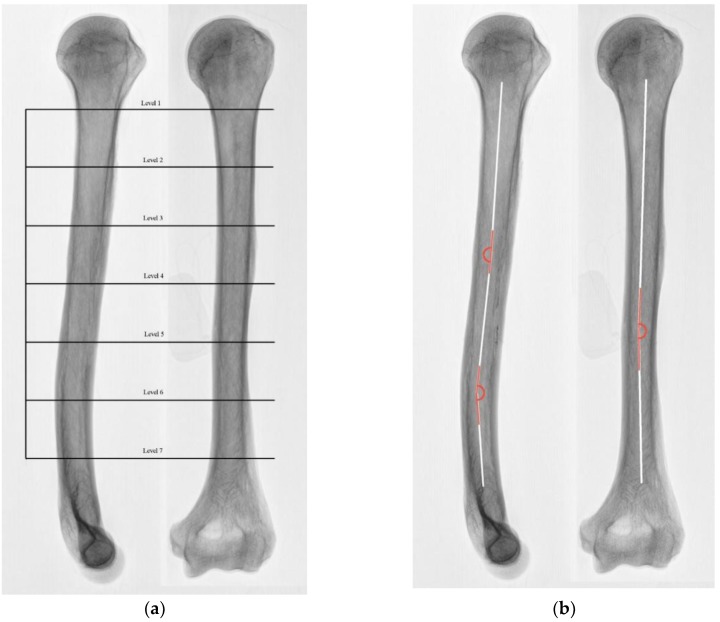
Left humerus, lateral (left) and anteroposterior (AP) radiograph (right). (**a**) Measurement levels from the surgical neck to the beginning of the medial supracondylar ridge are marked in black as 1–7. (**b**) The axis of the medullary canal is marked in white, and the corresponding angles are marked in red. While the slight valgus bending seems irrelevant, pro- and especially recurvatum angles should be considered both in ante- and retrograde nailing.

All specimens of the anatomical evaluation originated from voluntary body donations to the Division of Anatomy and Cell Biology. Twenty formaldehyde–phenol embalmed specimens (8 male, 12 female) without any evidence of humeral fractures, prostheses, osteomyelitis, or nailing were included in this evaluation. Samples were randomly selected based on the availability at the division for Anatomy and Cell Biology. Bare bones were packed in sterile vacuum bags and prepared for radiological evaluation. Anteroposterior (AP) as well as lateral radiographs were carried out and millimeter scales were added to each radiograph for exact measurements. For further dissection, each humerus was oriented with the epicondyles lying flat on the table surface and the anterior margin facing up on the preparation table. All sections were performed using the EXAKT 312 Diamant Band Pathology Saw (2019 EXAKT Technologies, Inc., Oklahoma City, OK, USA). The forefront along the intertubercular groove and the anterior margin were marked. The length of the medullary cavity was measured in the radiographs from the surgical neck to the supracondylar fossa, and this was divided into 7 parts (Figure 1a). A similar method has already been published in the literature [12]. For this study, the procedure was adapted and improved by taking regular measurements at constant instead of random distances. At the defined and marked levels, horizontal sections of the bones were taken with the pathology saw. A sliding caliper with an accuracy of 0.03 mm was used to measure the sagittal and coronal diameters of the sections as well as anterior, posterior, medial, and lateral CoTs. This paralleled the approach taken for our radiological measurements; at the previously marked levels, the coronal and sagittal diameters of the humeral cavity and CoT were measured using ImageJ software [14]. These anatomical and radiological results were then compared and analyzed.

### 2.3. Statistical Analysis

Statistical analysis was performed with IBM SPSS Statistics software (BM Corp. Released 2018. IBM SPSS Statistics for Windows, Version 25.0. Armonk, NY, USA: IBM Corp.). Descriptive statistics (mean, SD, minimum and maximum) were computed for all metric variables. The course of the medullary canal and the alteration of the medullary cavity width (MCW) and CoT from proximal to distal points were measured. The maximum and minimum medullary MCW and CoT were determined. Furthermore, differences in sex, arm dominance, and age were identified. The intraclass correlation κ was used for the comparison of radiographic and anatomical measurements in group 2 (≥0.9 strong; 0.75–0.9 good, 0.75–0.5 weak, <0.5 weak) [15]. The Pearson coefficient *r* was used for the correlation of sex and age (>0.8 < *r* ≤ 1.0 strong; 0.5 < *r* ≤ 0.8 moderate; 0.2 < *r* ≤ 0.5 weak; 0.0 < *r* ≤ 0.2 no correlation). For normally distributed metric data, a Student’s *r*-test was used, while non-normally distributed variables were analyzed using a Mann–Whitney U test. For categoric variables, the chi-square test was performed. A *p*-value < 0.05 was considered significant. The Bonferroni correction was applied for multiple testing.

## 3. Results

Thirty humeri from 25 individuals were included in this study (15 female, 15 male, 16 left, 14 right). The mean age of the included specimens was 70.13 ± 22.49 (18.00–100) years. In group 1 (clinical patients), the mean age was 49.5 ± 24.39 years (18.00–84.00), while the mean age in group 2 (anatomical specimens) was 80.45 ± 12.41 years (58.00–100.00). All specimens showed an appropriate bone quality, and there was no sign of recent or healed fractures. No degenerative changes were found along the meta- and diaphysis.

### 3.1. Medullary Cavity Width (MCW)

The mean results for all specimens are shown in Table 1. For all groups, the largest diameter was seen at level 1 in the coronal orientation, and the smallest diameter was observed at level 7 in the sagittal orientation. The mean diameter ranged from 21.88 mm in the coronal orientation at level 1 (surgical neck) to 10.15 mm in the sagittal orientation at level 7 (supracondylar). At levels 1 and 2, the smallest diameters were found in the sagittal orientation. At level 4, the sagittal diameter became larger than the coronal diameter (13.36 vs. 12.50). This proportion remained until level 7, where, once again, the sagittal diameter became the smallest dimension.

There was a difference between female and male humeri proximally, but no difference was observed after level 2. The difference in MCW was statistically significant at level 1 in the coronal (*p* = 0.014) and sagittal (*p* = 0.001) orientations, and at level 2, in the sagittal (*p* = 0.011) orientation. There were no statistically significant correlations between age, humerus length, and MCW.

The comparison of anatomical and radiological measurements of group 2 (anatomical specimens) showed different results (Table 2). X-ray measurements of the MCW regularly displayed greater values than the true anatomical ones. The intraclass correlation revealed good to excellent results for levels 1 to 4, but only moderate correlations for levels 5 to 7. A particularly poor correlation was found at level 7 in the sagittal orientation. Radiographic measurements overpredicted values by 7.8% at the proximal, 12.6% at the midshaft, and 24.5% at the most distal region of the humerus.

### 3.2. Cortical Thickness (CoT)

Detailed results of the smallest CoTs for each level are given in Table 1. From proximal to distal, CoTs showed increasing thickness, with the smallest mean values observed at level 1 and the largest at level 7.

Differences could be observed between female and male CoTs with the mean values for male CoTs being statistically larger on all measured levels (*p* < 0.001). There were statistically significant positive correlations between the anterior CoT and humerus length at level 6 (*p* = 0.002, *r* = 0.530) and level 7 (*p* = 0.005, *r* = 0.523), but no correlation was apparent between CoT and age. Apart from level 1, intraclass correlation for CoT revealed good to moderate results for the entire humerus (Table 3). No correlation was shown for level 1. The intraclass correlation for anterior CoT showed good results for level 6 (κ = 0.867) and moderate results (κ = 0.675) for level 7.

### 3.3. Course of the Humeral Cavity

AP and lateral radiographs illustrated the alteration of the humeral cavity over the course of the humerus. (Figure 1b). In the AP view, minimal valgus bending was noted, and the lateral view demonstrated an S-shaped course. The mean angles of the pro- and recurvatum of the investigated specimens were 4.01 ± 1.68° and 10.03 ± 2.25°, and the mean valgus bending was 3.37 ± 1.58°. There were no differences between male and female specimens and no age correlation.

## 4. Discussion

Intramedullary humeral lengthening has become more and more common in recent years [7,16]. While safety spots for intramedullary nailing and locking screws are well-known in clinical practice, the shape of the humeral cavity has been neglected as an important parameter in preoperative planning [17,18]. Our aim, therefore, was to investigate the anatomical direction of the medullary cavity, which is important when implanting straight nails during fracture fixation and lengthening.

The most important findings of the study were that the smallest MCWs were seen at the distal humerus, and they decreased gradually from proximal to distal points, as shown in Table 1. Furthermore, female CoTs were significantly smaller on all levels (*p* < 0.001). The correlation of our radiographic and anatomical measurements revealed moderate to good results. However, X-ray measurements generally overpredicted the MCW by 7.8% at the proximal, 12.6% at the diaphyseal, and 24.5% at the most distal part, whereas the CoT was generally underpredicted by 13% without a specific predilection site. Even smaller MCWs and larger CoTs were observed in our CT group. This could be attributed to the younger age of our CT group, as already observed by Ndou et al. [11]. In our study, recurvatum with a mean value of 10.03 ± 2.25° was regularly found at the distal third of the diaphysis (levels 6 and 7, Figure 1b). Procurvatum with a mean value of 4.01 ± 1.68° was generally smaller and measured at the middle third of the diaphysis. No studies describing and quantifying these angles were found when PUBMED and SCOPUS were searched. All examined humeri further showed slight valgus bending with a mean angle of 3.37 ± 1.58° at the middle third.

Böhler et al. were the first to demonstrate the unique course of the humeral canal [10]. However, since then, there have only been a few anatomical studies that have indicated substantial differences among ethnicities. South Asians, for example, have a significantly narrower medullary cavity (7.2 vs. 9.1 mm) and thicker cortical bone than Arabs [12]. A correlation of higher body mass index (BMI) and increased cortical thickness was also published. Pospula et al. observed that the MCW was the smallest at the beginning of the distal third of the humeral canal [12]. However, their measurements were only taken in mediolateral orientations, limiting the validity of their conclusions. Comparing these results with our coronal measurements showed similar results. Our study, which used samples from Caucasian patients, placed its measurements squarely between the results previously published for Arabs and South Asians. Murdoch et al. reported quite different results in terms of MCWs, averaging 12.1 ± 2.6 mm, without stating a specific level of measurement [19]. Ndou et al. compared the MCW and CoT at three points along the diaphysis using MRI scans [11]. A strong negative correlation between these two variables was observed. However, in this study, measurements were only taken in the mediolateral position. They concluded that the MCW depends on the humeral bone size, which was not observed in our study. We did not see any correlation between humerus size and MCW, although we observed a correlation between humerus size and CoT.

Two intramedullary implants for upper limb lengthening are currently available, both having a larger proximal and a smaller distal width. The PRECICE-UNYTE nail starts with a diameter of 10 mm at the proximal end, and it becomes narrower over its course with a diameter of 8.5 mm at the diaphyseal and 6.5 mm at the distal part [20]. In comparison, the thinnest FITBONE nail measures 11 mm at the proximal, 9 mm at the diaphyseal, and 6 mm at the most distal part [21].

**Figure 2:**
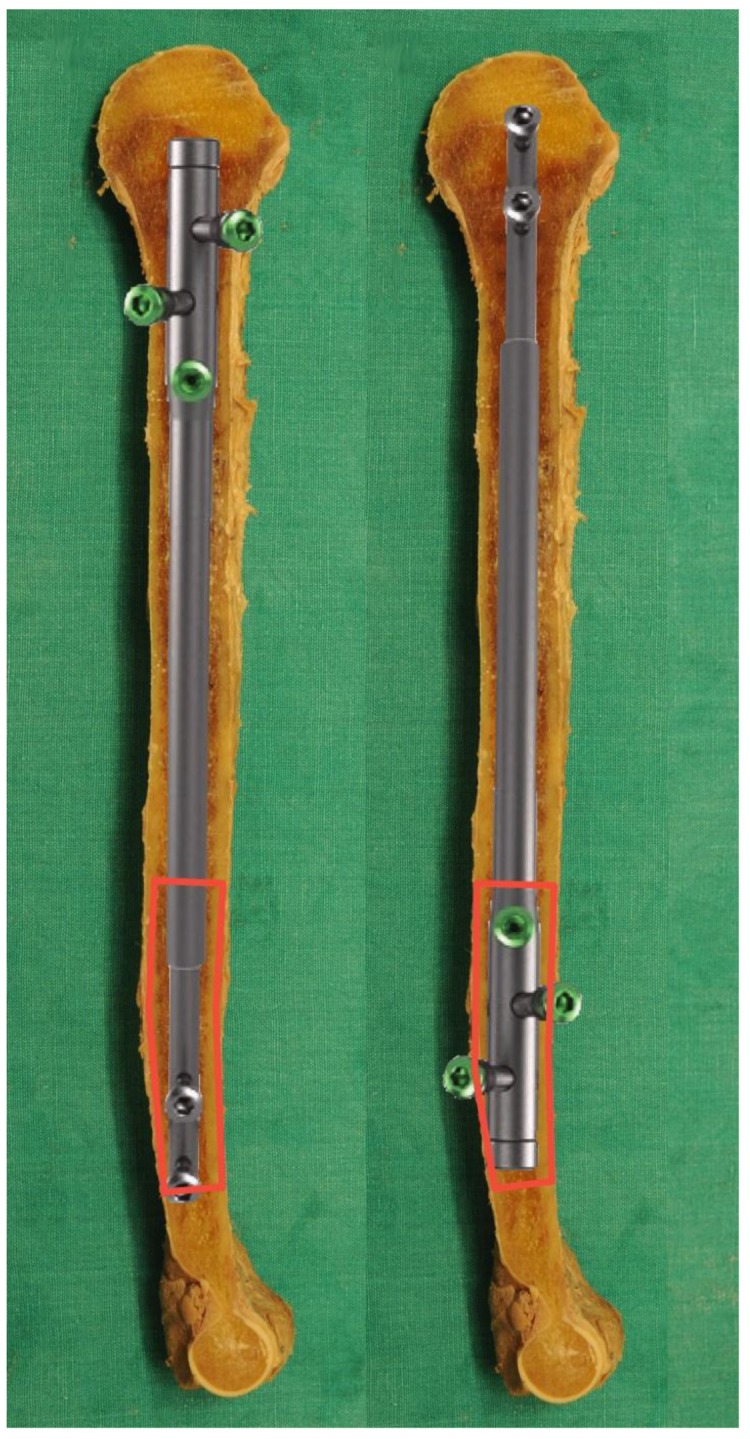
Left humerus, PRECICE UNYTE nail, comparison of ante-(**left**) and retrograde (**right**) nailing. Discrepancies in retrograde nailing of the MCW and nail sizes at the distal third of the humerus are demonstrated. The area at risk regarding iatrogenic fractures at the distal humerus.

Based on these anatomical findings, recent literature, and available implants, two main topics have to be considered. First, the MCW is not consistent. Although some typical ethnical characteristics exist, choosing the implant simply based on the patient’s origin might be dangerous as the humeral canal has a unique course [12]. Positive correlations between CoT, gender, and humerus size have to be considered, with females having significantly smaller CoTs over the entire course of the humerus (*p* < 0.001). Patients with post-traumatic injuries or congenital deformities could have even smaller bones. Second, from the diameters of available implants, the distal humerus with its small MCW appears to be the most critical zone in intramedullary nailing. Figure 2 clearly displays the anterior and posterior CoTs at levels 6 and 7, which are the most dangerous areas for iatrogenic fractures. These complications have been described frequently in retrograde nailing [4,8].

Therefore, exact preoperative planning is essential in humeral nailing and lengthening. AP and lateral radiographs of both humeri in combination with a measuring stick should be performed to define the humerus size and to select the correct dimension of the implant. Hereby, X-rays generally overpredict the MCW. CT scans of the distal third of the humerus are particularly helpful in preoperative planning, especially in female patients with smaller humeri. While valgus bending seems insignificant, the procurvatum of the medullary canal in the middle third is of significant concern. The S-shaped curvature may necessitate the reaming out of the corresponding internal curvature in two locations. Hereby, the already small CoT is further weakened and increases the risk of intraoperative fractures during nail insertion. Our study suggests that the implant should be chosen based on the given anatomical conditions, and the antegrade approach is the preferred and safer one.

As in all anatomical studies, the advanced age of the specimens and the small sample size are potential limitations of this study. In adding the results of CT scans from younger patients, we attempted to mitigate this limitation. Our relatively small sample sizes are attributed to the fact that we focused on general surgical considerations. For demographic comparisons, cross-sectional studies with higher numbers of specimens have to be performed. Correlations of body size and BMI could not be evaluated as these parameters were not gathered routinely in the anatomical department. In recent literature, CoT was only indirectly measured by subtracting the MCW from the overall bone width without a clear definition of measurement levels [11,12]. In our study, the MCW, CoT, and curvature of the humeral canal were individually measured at defined levels according to a strict study protocol. To the best of our knowledge, this is the first study describing the anatomy of the humeral canal in the context of intramedullary nailing and lengthening.

## 5. Conclusions

The unique course of the humeral cavity in combination with currently available implants makes exact preoperative planning of intramedullary humeral lengthening mandatory. We believe that, from an anatomical standpoint, antegrade nailing shows less fracture potential and is a safer approach for the S-shaped humeral canal.

## Figures and Tables

**Table 1:** Mean medullary cavity width (MCW) and cortical bone thickness (CoT) for all specimens (left) and for group 1 (right). Sag = sagittal orientation, cor = coronal orientation.

	MCW (Overall, *n* = 30) mm	CoT (Overall, *n* = 30) mm	MCW (Group 1, *n* = 10) mm	CoT (Group 1, *n* = 10) mm
Level 1 sag	21.31 ± 2.92	2.30 ± 0.43 ant 2.76 ± 0.44 post	19.19 ± 3.14	2.39 ± 0.64 ant 2.53 ± 0.92 post
Level 1 cor	21.51 ± 3.12	2.67 ± 0.40 med 2.23 ± 0.24 lat	21.76 ± 3.05	2.50 ± 0.71 med 2.03 ± 0.67 lat
Level 2 sag	14.94 ± 3.07	3.91 ± 0.76 ant 3.51 ± 0.71 post	14.93 ± 2.47	3.48 ± 1.02 ant 2.72 ± 0.83 post
Level 2 cor	15.05 ± 2.84	3.75 ± 0.73 med 3.29 ± 0.50 lat	15.84 ± 2.65	2.92 ± 0.92 med 2.58 ± 0.76 lat
Level 3 sag	12.82 ± 2.38	4.68 ± 1.13 ant 3.88 ± 0.64 post	13.86 ± 2.18	4.06 ± 1.30 ant 3.15 ± 1.09 post
Level 3 cor	11.54 ± 2.38	3.99 ± 0.75 med 4.52 ± 0.92 lat	13.92 ± 2.74	3.47 ± 0.93 med 3.98 ± 0.99 lat
Level 4 sag	12.30 ± 2.86	5.08 ± 1.20 ant 4.55 ± 0.67 post	13.36 ± 2.28	4.27 ± 1.43 ant 3.49 ± 1.04 post
Level 4 cor	9.91 ± 2.61	4.18 ± 1.09 med 4.08 ± 1.00 lat	12.50 ± 2.59	3.53 ± 0.91 med 3.94 ± 1.16 lat
Level 5 sag	10.37 ± 2.08	4.43 ± 0.68 ant 4.33 ± 0.49 post	12.80 ± 2.38	3.94 ± 1.02 ant 3.69 ± 1.05 post
Level 5 cor	9.32 ± 2.15	4.72 ± 0.72 med 4.75 ± 0.52 lat	11.32 ± 1.89	3.93 ± 1.15 med 3.58 ± 1.00 lat
Level 6 sag	9.25 ± 1.48	4.14 ± 0.41 ant 4.04 ± 0.51 post	12.01 ± 1.96	3.78 ± 0.99 ant 3.43 ± 1.03 post
Level 6 cor	9.64 ± 2.28	4.15 ± 0.42 med 4.04 ± 0.51 lat	11.35 ± 1.88	3.55 ± 0.96 med 3.68 ± 1.03 lat
Level 7 sag	8.93 ± 1.10	4.13 ± 0.45 ant 4.07 ± 0.45 post	10.15 ± 1.96	3.84 ± 1.02 ant 3.93 ± 1.15 post
Level 7 cor	9.93 ± 2.00	3.83 ± 0.58 med 4.25 ± 0.63 lat	11.66 ± 2.03	3.79 ± 1.09 med 4.29 ± 1.38 lat

**Table 2:** Mean MCW for group 2 and intraclass correlation between radiological and anatomical measurements (κ). Sag = sagittal orientation, cor = coronal orientation.

	MCW (Anatomical, *n* = 20) mm	MCW (Radiographs, *n* = 20) mm	Intraclass Correlation κ	Difference in %
Level 1 sag	17.59 ± 2.52	19.73 ± 3.12	0.778	12
Level 1 cor	20.50 ± 2.82	23.14 ± 2.78	0.754	12
Level 2 sag	14.77 ± 2.27	15.08 ± 2.45	0.961	2
Level 2 cor	15.68 ± 2.01	16.40 ± 3.09	0.768	4
Level 3 sag	13.98 ± 1.97	14.27 ± 2.21	0.915	2
Level 3 cor	13.48 ± 2.45	15.56 ± 2.17	0.761	15
Level 4 sag	13.05 ± 1.75	14.21 ± 2.24	0.763	8
Level 4 cor	12.14 ± 1.90	14.15 ± 1.98	0.649	16
Level 5 sag	12.52 ± 1.18	14.30 ± 2.36	0.590	14
Level 5 cor	10.97 ± 1.14	12.66 ± 1.23	0.565	15
Level 6 sag	11.79 ± 1.34	13.60 ± 1.52	0.618	15
Level 6 cor	11.31 ± 1.38	12.25 ± 1.55	0.849	8
Level 7 sag	9.00 ± 1.03	11.88 ± 1.72	0.231	32
Level 7 cor	11.13 ± 1.45	13.07 ± 1.65	0.506	17

**Table 3:** Mean smallest CoT for group 2 and intraclass correlation between radiological and anatomical measurements (κ).

	CoT (Anatomical, *n* = 20) mm	CoT (Radiographs, *n* = 20) mm	Intraclass Correlation κ	Difference in %
Level 1	2.35 ± 0.82 lat	1.60 ± 0.36 lat	0.113	46
Level 2	2.59 ± 0.63 post	2.11 ± 0.64 lat	0.616	22
Level 3	3.16 ± 1.05 post	2.77 ± 1.17 post	0.672	14
Level 4	3.16 ± 1.05 post	2.89 ± 0.92 post	0.722	9
Level 5	3.79 ± 1.07 post	3.03 ± 0.70 lat	0.683	25
Level 6	3.26 ± 0.99 post	3.25 ± 1.19 post	0.918	0
Level 7	3.74 ± 1.40 med	3.41 ± 0.89 ant	0.718	9
